# Facile Preparation Route of Cellulose-Based Flame Retardant by Ball-Milling Mechanochemistry

**DOI:** 10.3390/molecules29246065

**Published:** 2024-12-23

**Authors:** Mohamed Aaddouz, Fouad Laoutid, Jerome Mariage, Jevgenij Lazko, Bopha Yada, El Miloud Mejdoubi, Antoniya Toncheva, Philippe Dubois

**Affiliations:** 1 Laboratory Applied Chemistry and Environmental (LCAE), Faculty of Sciences of Oujda, Mohamed 1st University, Oujda 60000, Morocco; aaddouz.mohamed@ump.ac.ma (M.A.); ee.mejdoubi@gmail.com (E.M.M.); 2 Materia Nova Research Center, UMONS Innovation Center, Avenue Nicolas Copernic 3, B-7000 Mons, Belgium; jerome.mariage@materianova.be (J.M.); jevgenij.lazko@materianova.be (J.L.); bopha.yada@materianova.be (B.Y.); antoniya.toncheva@materianova.be (A.T.); philippe.dubois@umons.ac.be (P.D.)

**Keywords:** cellulose, flame retardancy, mechanochemistry, phosphorylation, bio-based

## Abstract

In this study, a sustainable cellulose-based flame-retardant additive was developed, characterized, and incorporated into polypropylene (PP). Microcrystalline cellulose (Cel) was chemically modified with P_2_O_5_ using the solvent-free ball-milling mechanochemistry approach at room temperature. This modification enabled phosphorus grafting onto cellulose, significantly enhancing the cellulose charring ability and improving the thermal stability of the char as revealed by thermogravimetric analysis (TGA). The resulting product, Cel-P, containing 4.15 wt.% phosphorus, was incorporated and uniformly dispersed as a flame-retardant (FR) additive at 30 wt.% in PP through melt processing. The PP+30-Cel-P composite demonstrated improved char formation and FR properties, including reduction of both peak heat release rate (pHRR) and total heat release (THR) in mass loss cone calorimetry (MLC). Moreover, lower light absorptivity was obtained by smoke opacity tests as compared to PP filled with unmodified cellulose.

## 1. Introduction

Today plastic materials are offering numerous advantages such as lightweight, easy processing, and reasonable cost, making them indispensable in various sectors such as transport (automotive, aeronautics, railways), sports, textiles, and construction [1]. However, these materials also present significant environmental challenges due to the origin of the raw materials required for their production and related end-of-life aspects. Most plastics are derived from fossil resources and are inevitably contributing to greenhouse gas emissions increase. Minimizing the ecological impact of plastics requires improved materials end-of-life management, primarily through efficient recycling [2] and the development of new materials from renewable resources, especially if integrated into circular economies [3].

Additionally, the durability of performance-enhancing additives used in plastics is crucial for their sustainable application. This is especially true for flame-retardant (FR) additives, which are incorporated to improve fire resistance and ensure that fire safety standards and technical requirements are met for each specific application [4].

Flame-retardant additives are designed to prevent polymer ignition, reduce heat release during combustion, and limit flame propagation by interfering with the physical or chemical processes involved in polymer combustion. Certain natural molecules and biopolymers can be effective in this application. For example, cellulose has an intrinsic ability to generate carbon during thermal degradation [5,6], although it requires enhancement to act effectively as a FR additive in polymers [7]. One of the most widely studied strategies is the formation of a char layer on the surface of the burning material. This barrier helps slow heat diffusion into the material and reduces the release of volatiles into the gas phase, thereby lowering the fuel supply to the flame.

Developing such FRs from renewable resources is a promising approach to supporting low-carbon footprint materials. Cellulose, the most abundant organic raw material in nature, shows significant potential as a bio-sourced FR additive due to its high carbon content and hydroxyl groups, which promote natural charring and enable chemical modification.

However, using cellulose alone does not yield sufficient FR properties, primarily because of its low resistance to thermo-oxidative degradation. Enhancing its thermal stability often requires combination with other FR additives (additive approach) [8,9], or, more effectively, chemically grafting of reactive molecules—typically phosphorus-containing—onto the cellulose (reactive approach) [8,10,11,12].

The reactive route also remains the most interesting one from an environmental point of view, as it usually enables a higher level of bio-based content to be achieved in the final material. However, this is only true if the chemical modification process used to treat cellulose is eco-friendly and has a low environmental impact; otherwise, the environmental advantage of using cellulose will be lost.

Traditional methods used for modifying cellulose often rely on corrosive, concentrated phosphoric acid or derivatives; require purification steps that consume large amounts of organic solvents; and involve energy-intensive drying techniques.

The development of milder and more environmentally friendly cellulose phosphorylation processes has become essential. Lee et al. demonstrated cellulose phosphorylation via an enzyme-based process, utilizing hexokinase and adenosine-5’-triphosphate (ATP) in the presence of magnesium ions on cellulose nanofibers [13].

Over the past decade, mechanochemistry has emerged as a valuable tool, offering the possibility of achieving chemical transformations with minimal waste and often without any solvent. This method involves initiating chemical reactions through the grinding of reagents [14,15]. Ball milling is one of particularly efficient mechanochemical processes, as it is cost-effective, is straightforward to implement, and enables the processing of large volumes. The rapid rotation of the balls generates shear forces and increases the temperature of the reaction mixture [16,17]. However, solid-state mechanochemistry also presents certain limitations, particularly when it comes to modifying thermoplastic polymers. These materials tend to soften and melt under heat, causing solid-state milling to primarily result in the absorption and dissipation of mechanical energy. As a consequence, the process fails to generate the specific local temperature and pressure effects required for efficient modification [18].

Given the limited examples of large-scale processes available in the literature, synthetic mechanochemistry has often been overlooked due to a lack of evidence at the manufacturing scale. However, equipment enabling rapid scale-up is already available. For instance, we can cite the eccentric mills and horizontal high-energy ball mills by Simoloyer^®^ (https://www.siebtechnik-tema.com) and Eccentric Mill (https://www.continuumtech.ca, accessed on 10 December 2024), which can process up to 200 L and 1522 L, respectively. Nonetheless, it must be acknowledged that scaling up is far from a linear extrapolation of small-scale research; it requires more than simply employing larger equipment [19].

Recently, mechanochemical phosphorylation has been used to modify cellulose nanofibers, aimed at producing paper with enhanced fire resistance [20,21]. In both studies, phosphorylation was successfully achieved using phosphorus pentoxide (P_2_O₅) in a ball mill. P_2_O₅ was preferred because it did not produce any by-product, unlike chlorinated phosphorus agents for example, which release HCl during their grafting. However, the presence of P_2_O₅ in contact with cellulose can also impact it and cause some structural modifications, which should be carefully avoided. In fact, P_2_O_5_ can be mix-milled with cellulose for breaking its crystalline structure and increasing glucose yield during the chemical hydrolysis process [22].

In our study, we developed a phosphorylated cellulose (Cel-P) from microcrystalline cellulose by ball-milling mechanochemistry. The Cel-P product was purified by several washing/centrifugation steps in water and characterized by thermogravimetric analysis (TGA), Fourier transform infrared spectroscopy (FTIR), inductively coupled plasma (ICP) analysis, and scanning electron microscopy–energy dispersive X-ray analysis (SEM-EDX). Unmodified cellulose (Cel) and phosphorylated cellulose (Cel-P) were finally blended into PP at 30 wt.% by melt processing. The effect of their incorporation on the thermal and flame retardant properties of PP composites was studied using TGA, cone calorimetry, and smoke opacity tests.

## 2. Results and Discussion

### 2.1. Cellulose Modification

#### 2.1.1. Structural Characterization

The treatment of cellulose with P_2_O_5_ in the ball mill was designed to graft phosphoric acid onto the surface of microcrystalline cellulose microparticles (see experimental). ICP analysis of Cel-P indicated a phosphorus content of 4.15 wt.%, indicating that approximately 33% of the phosphorus initially introduced into the blend was successfully grafted. The phosphorus grafting rate achieved through mechanochemistry appeared lower compared to the relative content obtained via a more conventional solvent-based process. For instance, using phosphonic acid and urea, the P content reached approximately 16.5 wt.% [8], while employing phosphoric acid and aqueous ammonia resulted in a P content of 22.51 wt.% as determined by X-ray photoelectron spectroscopy [12]. This difference was most likely due to the specificity of the phosphorus-grafting mechanisms. In the ball-mill process, grafting occurs mainly on the surface of the cellulose microparticles, whereas in the protocol using urea and phosphoric acid, cellulose is solubilized, allowing functionalization to occur directly on unfolded cellulosic molecular chains. However, it is important to highlight that the phosphorus content achieved in our study was higher than what has been reported for other solvent-based modification reactions. For instance, in a study by Yuan et al. on the modification of microcrystalline cellulose using phytic acid, a maximum phosphorus content of only 0.630 wt.% was achieved [23].

Quantifying the P content by ICP enables us to evaluate the average number of substituent groups attached per monomer unit, known as the degree of substitution (DS). DS of cellulose by grafted phosphorous groups can be estimated using the empirical formula [24].

(1) $$DS=\frac{Mcellulose\times P\%}{100\times M\left(P\right)-\Delta M\times P\%}$$

M_cellulose_ is the molecular weight of cellulose D-glucose monomer unit (162 g/mol), P% is the weight % of phosphorus in modified cellulose (4.15 wt.%), M(P) is the molar mass of phosphorus (31 g/mol), and ΔM is the molar mass of substituent—molar mass of leaving group (M(H_3_PO_4_) – M(H) = 97 g/mol).

According to this calculation, a degree of substitution of 0.25 was obtained. In comparison, phosphorylation cellulose with phosphonic acid and urea as catalysts at 150 °C for 2 h yielded a DS of 1.3 [8].

**Figure 1:**
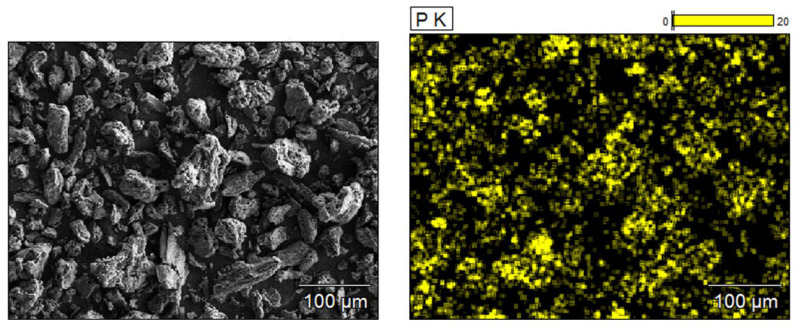
SEM-EDX micrographs showing the map of distribution of P on the surface of Cel-P. SEM image of Cel-P particles (**left**) and map of distribution of P on the surface (**right**).

The analysis of phosphorus content in Cel-P was complemented by an assessment of phosphorus localization on its surface. To this end, SEM-EDX analyses were carried out for mapping the atomic density of P. Figure 1 clearly evidences the distribution of P along the entire surface of modified cellulose (Cel-P), demonstrating the effectiveness of the process used to functionalize the cellulose surface.

**Figure 2:**
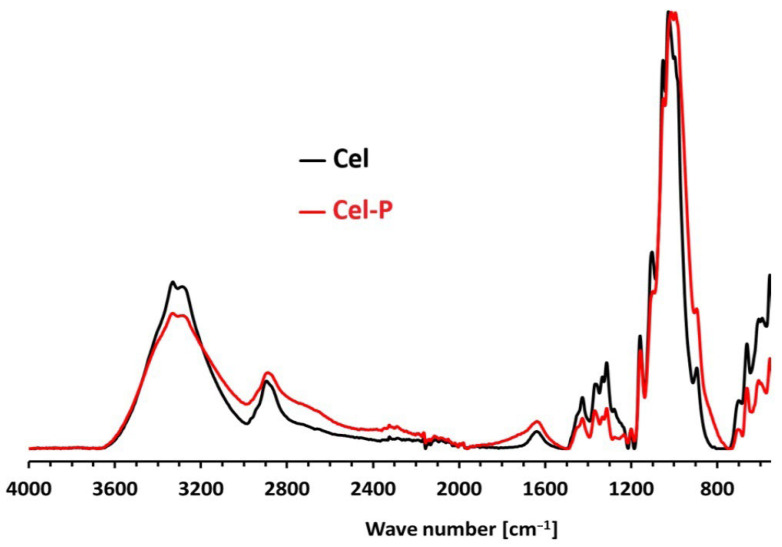
FTIR spectra of Cel and Cel-P.

Despite confirmation of phosphoric acid grafting on the cellulose surface by both SEM-EDX and ICP, FTIR spectra (Figure 2) showed no fundamental difference between Cel and Cel-P. A shoulder at around 850 cm^−1^ could be assigned to the new characteristic C-O-P bonds. This slight difference was mainly due to the low grafting rate of phosphoric acid, with a DS of only 0.25.

#### 2.1.2. Thermogravimetric Degradation

**Figure 3:**
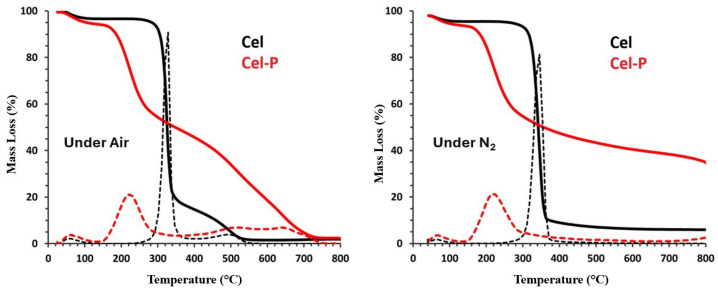
TGA (continuous curves) and DTG (dotted curves) of Cel and Cel-P, under air and under N_2_, at 10 °C/min. TGA (continuous curves) and TGA.

In order to assess the effect of phosphorylation on cellulose thermal stability, TGA was performed under air and N_2_ on both Cel and Cel-P. Weight loss curves vs. temperature and data are represented in Figure 3 and Table 1, respectively. The degradation profiles of the two celluloses are highly dependent on the nature of the atmosphere used. Under N_2_, both samples underwent thermal degradation in a single step, whereas in air, the DTG curves displayed two distinct decomposition peaks. We can also note a slight mass loss below 100 °C, corresponding to a minor weight reduction (<5 wt.%), which is attributed to the evaporation of water adsorbed on the surface of both cellulose samples in both atmospheres.

Modification of cellulose with P_2_O_5_ in the ball mill induces a change in its thermogravimetric degradation pathway. As can be seen from the thermograms, the Cel exhibited a better thermal stability than the modified one in both atmospheric conditions. In fact, the temperature at which unmodified cellulose displayed 20 wt.% weight loss was around 305 °C under air and 328 °C under N_2_, whereas it was only 215 °C for phosphorylated cellulose, whatever the nature of the gas. Moreover, the degradation of Cel-P began at around 160 °C, which limited the range of polymers in which it can be melt-processed. In our case, the composites were processed at 180 °C, meaning that a slight degradation of Cel-P during mixing cannot be entirely ruled out.

The low thermal stability of Cel-P is attributed to the presence of phosphoric-acid-grafted groups, which was already reported to catalyze cellulose dehydration, internal rearrangement reaction, and the formation of dehydrated cyclic char structures [25,26]. These effects account for the improved thermal stability of the residue observed above 400 °C. This enhancement is particularly advantageous as it provides the modified cellulose with effective flame-retardant properties. During combustion, a protective char layer forms, acting as a barrier that reduces the release of flammable volatiles into the gas phase and minimizes heat transfer through the material. In fact, even if Cel started to degrade later, it decomposed very rapidly to reach completion at 550 °C under air, while only 6% remained at 400 °C under N_2_. In contrast, the amount of residue formed during thermal decomposition of Cel-P was significantly greater. Under air, the second degradation stage, corresponding to the degradation of the residue, occurred more slowly, and the amounts of residue at 340 °C and 450 °C were about 50% and 40%, respectively, for Cel-P, and only 20% and 10%, respectively, in the case of unmodified cellulose. The phenomenon was even more pronounced under air, with Cel degrading almost completely (6%) at 800 °C, while a residue of 35% remained with Cel-P.

Phosphorylation of cellulose induced a fundamental change in its thermogravimetric behavior. On the one hand, the presence of phosphoric acid units induced premature thermal degradation from around 215 °C, and on the other, it led to an improvement in its capacity to form chars in greater quantities and with better thermal resistance.

### 2.2. PP Composite Properties

#### 2.2.1. Morphology and CEL-P Dispersion

**Figure 4:**
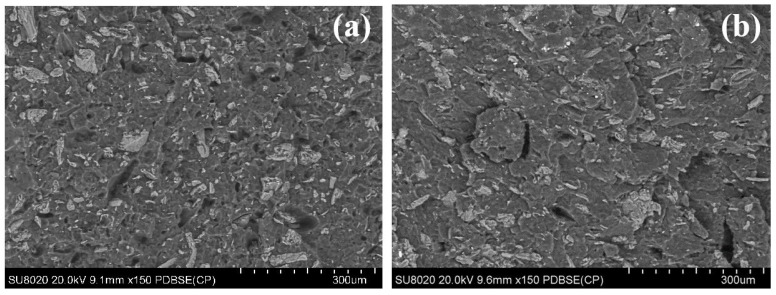
SEM micrographs of cryo-fractured surface of (**a**) PP+30-Cel and (**b**) PP+30-Cel-P composite, acquired in chemical contrast mode.

**Figure 5:**
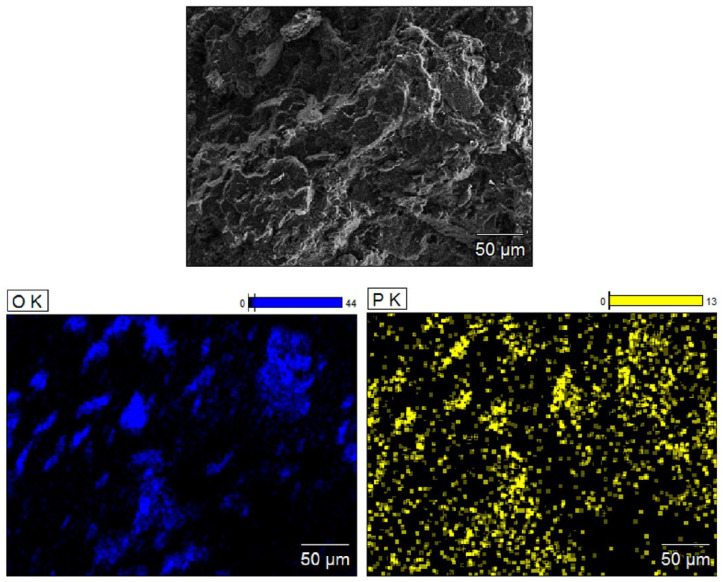
SEM (Top) and SEM-EDX (down) micrographs of the cryo-fractured surface of PP+30-Cel-P composite, showing the dispersion of oxygen (on the **left**) and phosphorus elements (**right**).

Cel and Cel-P were incorporated into PP at 30 wt.%. Ensuring proper dispersion of the Cel-P particles within the polymer matrix was essential, as poor dispersion could hinder the FR effect of the additive, thereby compromising the composite final performance. SEM micrographs, acquired in chemical contrast mode, highlighting heavier elements such as oxygen and phosphorus (appearing brighter), are shown in Figure 4. These images clearly demonstrate that (1) ball milling did not impact the particle size, as the particle sizes of Cel and Cel-P in both composites remained comparable, and (2) both Cel and Cel-P particles were well-dispersed throughout the matrix. This finding is further supported by the SEM-EDX micrographs (Figure 5), revealing a good distribution of oxygen and phosphorus elements, characteristic for Cel-P.

#### 2.2.2. Thermogravimetric Properties

**Figure 6:**
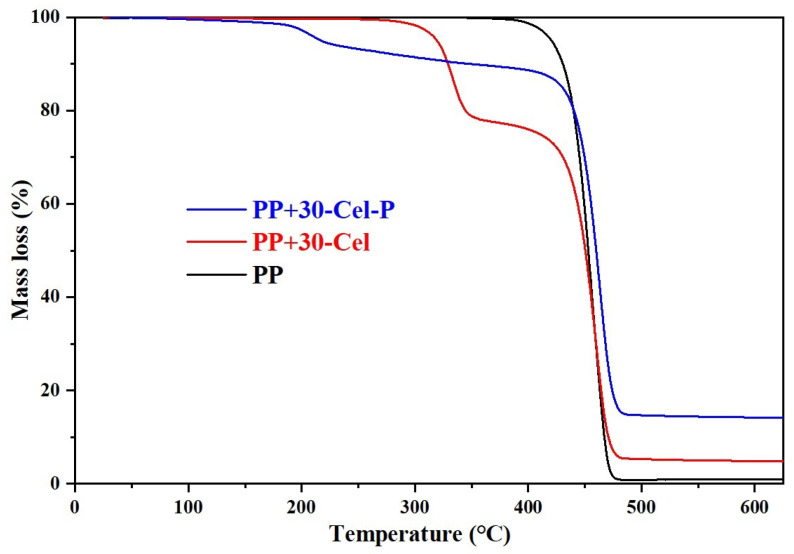
TGA thermograms of neat PP and composites containing 30 wt.% Cel and Cel-P, under air at 10 °C.min^−1^.

The incorporation of cellulose, regardless of its chemical functionalization, significantly altered the thermogravimetric behavior of PP (Figure 6). While neat PP degraded completely in a single step, the degradation of PP–cellulose composites occurred in two stages, with the process initiating at lower temperatures. The first stage likely corresponded to the degradation of cellulose and its derivatives, while the second stage can be attributed to the degradation of PP and the residue formation. The temperature at which thermal degradation begins was therefore dictated by the thermal degradation characteristics of the cellulose used. The initial degradation stage occurred at a lower temperature with Cel-P (218 °C) than with unmodified cellulose (330 °C), closely aligning with the previously described thermogravimetric behavior of the two types of cellulose (Figure 3). However, the degradation rate was slower in the presence of Cel-P, as 20% and 40% mass losses occurred at higher temperatures with Cel-P. Specifically, T_-20%_ and T_-40%_ were at 440 °C and 455 °C for PP+30-Cel-P, compared to only 344 °C and 445 °C for PP with unmodified cellulose. This result aligns well with TGA analyses of the two celluloses alone (Cel and Cel-P), highlighting a slowing in degradation rate after the initial stage due to the increased carbonization potential of Cel-P. This same phenomenon also accounted for the enhanced thermal stability of the PP+30-Cel-P composite, which produced a higher amount of residue at the end of the test, reaching 13% at 800 °C, compared to only 5% with unmodified cellulose. Even a small content in phosphorus grafted onto the cellulose significantly improved its thermogravimetric behavior and that of the composite.

#### 2.2.3. Cone Calorimetry

The mass loss cone calorimeter (MLC) test was used for evaluating the effect of cellulose chemical modification on the fire behavior of PP composites. MLC provides the heat release rate (HRR) curve and allows the measurement of various combustion-related parameters, including time to ignition (TTI), peak of heat release rate (pHRR), total heat release (THR), and mass loss during combustion.

**Figure 7:**
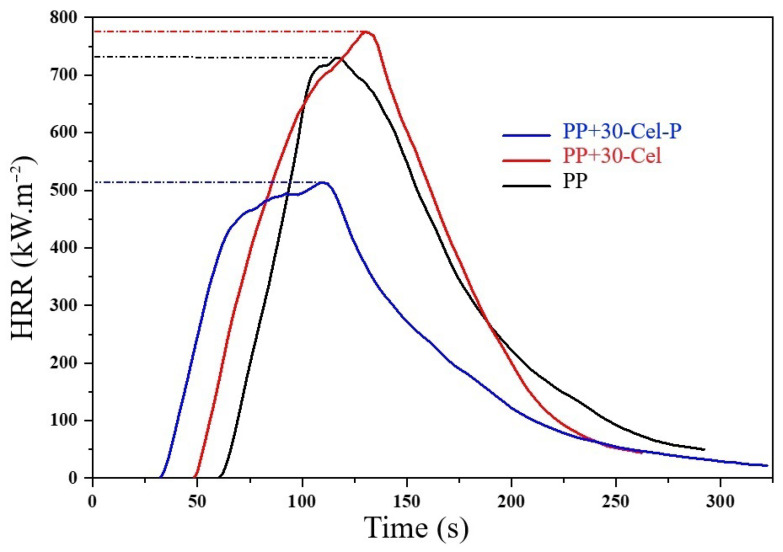
HRR curves of neat PP and PP composites, obtained by the mass loss cone calorimeter test at 35 kW.m^−2^.

**Figure 8:**
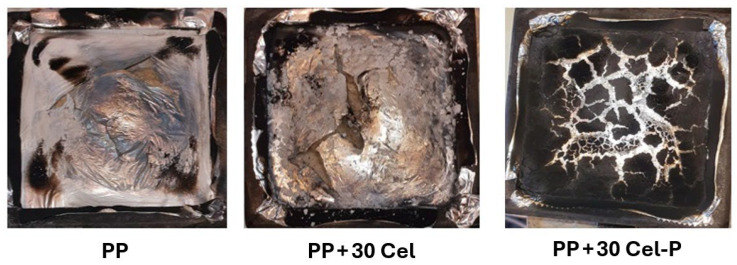
Images of char residues after MLC test of PP and cellulose-based composites.

The HRR curves for PP and its composites containing either Cel or Cel-P at 30 wt.% are compared in Figure 7. The key parameters of the fire retardancy of the PP composite values were extracted and are provided in Table 2. The HRR vs. time curve recorded when testing PP alone shows that this polymer burned quickly and completely, with an ignition at 60 s accompanied by a high pHRR of 725 kW.m^−2^ and no residue formation (Figure 8).

It is also worth mentioning that the incorporation of Cel resulted in a reduction of composite resistance to ignition down to 50 s and to any enhancement in pHRR that remained high (774 kW.m^−2^), both due to the combustible nature of cellulose. Cellulose alone, although capable of forming a residue during pyrolytic degradation (Figure 3), did not provide any protection for PP during combustion. The use of phosphorylated cellulose is recommended for this type of application, as it forms a more robust char resistant to thermo-oxidative degradation, as already demonstrated by TGA for both Cel-P (Figure 3) and PP+30-Cel-P composite (Figure 6).

Replacing Cel with Cel-P effectively reduced the pHRR down to 514 kW.m^−2^, achieving a 30% reduction. This improvement was attributed to the formation of a protective char layer during combustion, which limited the release of combustible volatiles into the gas phase. However, this residue decomposed almost entirely by the end of the test, leaving only small fragments as remnants of the combustion process (Figure 8). The use of phosphorylated cellulose also brought about an additional improvement by reducing the total heat release (THR) (Table 2). When unmodified cellulose was used, THR increased from 74.1 MJ.m^−2^ for PP up to 79 MJ/m^2^ PP+30-Cel, but decreased to 62 MJ/m^2^ for the composite containing Cel-P. Despite these improvements, an even more pronounced reduction in the ignition resistance of the composite was observed with Cel-P, as shown by a decrease in the TTI to 34 s. This behavior is linked to the reduced thermal stability of phosphorylated cellulose and corresponding PP composite, as indicated by TGA, due to the catalytic effect of the phosphoric acid groups.

The use of Cel-P provided notable improvements in the fire behavior of PP but reduced its ignition resistance. Combinations with other biobased FR, such as phosphorylated lignin [27], could help mitigating this negative effect and even enhancing performance.

#### 2.2.4. Smoke Density

**Figure 9:**
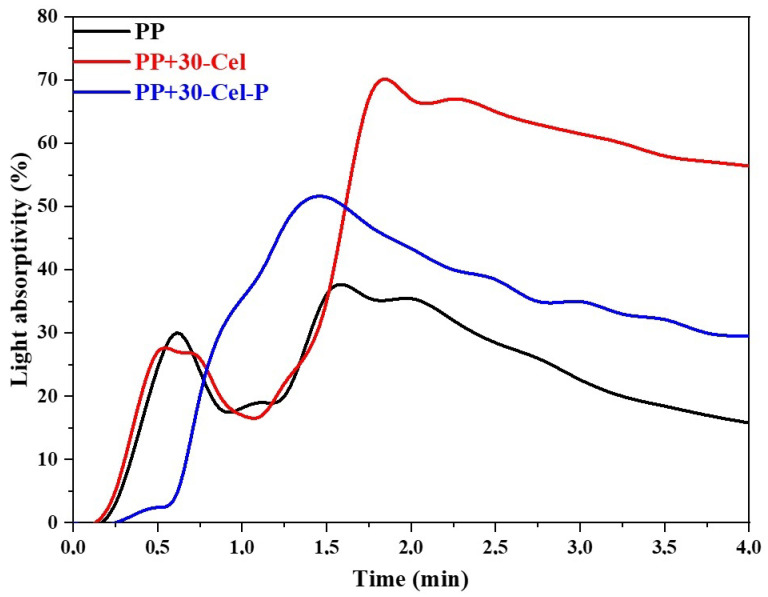
Light absorptivity curves of neat PP, PP+30-Cel, and PP+30-Cel-P composites.

The effect of incorporating Cel and Cel-P on the density of smoke during combustion of PP composites was evaluated in a smoke chamber following ASTM D2843 [28]. In fact, it is very important to consider this parameter that also represents a critical element of fire safety. Curves of light absorptivity vs. time are shown in Figure 9.

The combustion behavior of the three materials differed significantly. PP alone produced the least smoke, which can be attributed to three main factors: (1) PP combustion is not impeded by the presence of FR additives, allowing it to proceed toward complete combustion; (2) PP ignites quickly, leading to combustion of the fumes in the chamber and thus the reduction of their amount, which accounts for the initial peak observed between 0.5 and 0.75 min; and (3) PP tends to drip, meaning that a substantial portion remained unburned during the test, causing the secondary peak after 1.5 min.

In the case of the PP+30-Cel composite, ignition of the material led to the appearance of the first peak, as in the case of PP, except that the absence of droplets led to further smoke production, more so than in the case of PP. In addition, the contribution of cellulose has to be added, which also contributes to smoke production, reaching very high levels (70%).

On the other hand, composites containing Cel-P showed a totally different behavior. The absence of the first peak resulted from the absence of material ignition. When continuously exposed to flame, the material neither ignited nor dripped. The absence of ignition, combined with the formation of a char, means that the amount of smoke continued to increase until it reached around 50%, then decreased slightly due to the sporadic appearance of a few small flames and the evacuation of a little smoke from the bottom of the apparatus.

In summary, the results of these tests highlight a superior effect of the PP+30-Cel-P composite, despite its tendency to produce denser smoke as compared to neat PP. While the smoke opacity was higher than that of PP, it remained lower than that observed for PP blended with unmodified cellulose. Notably, this increased opacity was mainly due to the absence of droplet formation. In contrast, the lower smoke level observed in neat PP resulted from two adverse effects: gas ignition and droplet formation. Furthermore, the presence of phosphorus-based FR, specifically Cel-P in our case, aims to disrupt the fire triangle and hinder combustion, leading to incomplete combustion that generates more smoke than when the polymer burns alone.

## 3. Materials and Methods

### 3.1. Materials

Polypropylene (PP) used in this study is a high-impact-modified copolymer (PP 402 CB12) adapted for injection-molding applications, provided by INEOS Olefins & Polymer Europe (Cologne, Germany). This grade is characterized by a melt flow rate (230 °C, 2.16 kg) = 12 g/10 min, tensile strength at yield = 25 MPa, and Izod impact strength = 6 kJ.m^−2^ and 10 kJ.m^−2^ (at −20 °C and +23 °C, respectively). Microcrystalline cellulose and phosphorus pentoxide (P_2_O_5_, purity 99%) were purchased from Sigma Aldrich (Burlington, MA, USA).

### 3.2. Preparation of Phosphorylated Cellulose

Cellulose was chemically modified with P_2_O_5_ through ball milling in a PM 400 planetary mill (Retsch, Haan, Germany). A mixture of 20 g microcrystalline cellulose and 26 g P_2_O_5_ was introduced in a 500 mL stainless steel jar along with 60 stainless steel balls and processed at room temperature and 200 rpm. The milling was set for one hour, with a 30 sec pause every 10 min. We set the ball-milling time to 1 h to allow sufficient time for the grafting reaction to occur while minimizing the risk of cellulose degradation during the process. Indeed, Liu et al. [22] demonstrated that the degree of polymerization (DP) of cellulose decreases more rapidly during ball milling in the presence of P_2_O_5_, with a significant drop after 1 h of treatment at 500 rpm. Cellulose degradation during ball milling leads to the formation of water-soluble oligomers.

**Scheme 1:**
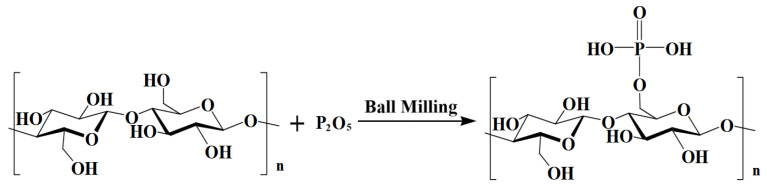
Schematic representation of the reaction between cellulose and P_2_O_5_.

After milling, the resulting Cel-P mixture was exposed to air until slightly pasty, then rinsed with cold deionized water to remove any unreacted phosphorus and eventual cellulosic water-soluble oligomers, which dissolve in water. The mixture was then washed/centrifuged 4 times at 4 °C at 10,000 rpm for 10 min using a refrigerated benchtop centrifuge Sigma 6-16KS) from Sigma Laborzentrifugen GmbH (Osterode am Harz, Germany). Finally, the recovered powder, i.e., phosphorylated cellulose, was dried in an oven at 40 °C. Scheme 1 illustrates the possible reactions during ball milling between cellulose and P_2_O_5_.

### 3.3. Composite Preparation by Melt Compounding

The composite preparation was performed using a Brabender bench-scale kneader (Brabender GmbH & Co. KG, Duisburg, Germany) at 180 °C, with an initial mixing phase of 3 min at 30 rpm, followed by 7 min at 90 rpm. Both PP and additives were pre-dried overnight in an oven at 60 °C. The 100 × 100 × 3 mm^3^ and 50 × 50 × 3 mm^3^ plates for cone calorimetry and smoke density measurements, respectively, were molded using an Agila PE20 hydraulic compression press (Menen, Belgium) at 180 °C. The sample was first placed on the heated section for 3 min, then pressed for 1.5 min at 50 bar, followed by three degassing steps, then pressed again for 3 min at 150 bar, before being transferred to the cold section for pressing over 5 min.

### 3.4. Characterization and Testing

Inductively coupled plasma (ICP) analysis was used for the determination of phosphorus content in the modified cellulose and was performed with an Optima 7300dV ICP–OES instrument from Perkin Elmer (Waltham, MA, USA). Prior to ICP analysis, the sample was digested using nitric acid. The content of phosphorus was determined using calibration curves obtained from ICP analyses of standard solutions at different concentrations (5 and 10 ppm) in combination with unmodified cellulose.

FTIR spectra were recorded using a Bruker ALPHA II spectrometer (Bruker Optics, Ettlingen, Germany), in the wavenumber range from 4000 cm^−1^ to 400 cm^−1^, with 2 cm^−1^ resolution and an accumulation of 32 scans.

Thermal stability of Cel, Cel-P, PP, and related composites was studied by TGA using TGA II equipment from Mettler Toledo (Greifensee, Switzerland). Approximately 5 mg of the sample was subjected to a temperature ramp from 30 °C to 800 °C at a heating rate of 10 °C.min^−1^ under both air and N_2_ in the case of cellulose samples and only under air for composites. The decomposition temperatures corresponding to 20% (T_-20%_) and 40% (T_-40%_) mass loss, onset thermal degradation, and residual weights at 800 °C were determined.

Scanning electron microscopy–energy dispersive X-ray analysis (SEM-EDX) was used to evaluate the dispersion of phosphorus in Cel-P, as well as through PP+30-Cel-P composite. Analysis was performed using an SU-8020 Hitachi instrument (Hitachi, Tokyo, Japan).

Fire behavior of PP and PP composites was evaluated through a mass loss cone (MLC) from Fire Testing Technology (East Grinstead, West Sussex, UK) following ISO 13927 standard (https://www.iso.org/standard/82203.html accessed on 19 November 2024). Samples (100 × 100 × 3 mm^3^) were exposed to an external heat flow of 35 kW/m^2^. Key metrics, including peak heat release rate (pHRR), total heat release (THR), and time to ignition (TTI), were recorded to analyze fire performance.

Smoke density in the PP samples was measured according to ASTM D2843 (https://www.astm.org/d2843-22.html accessed on 19 November 2024). Specimens (50 × 50 × 3 mm^3^) were placed on a metal screen in a test chamber and exposed to an open flame under 276 kPa pressure, using a propane burner for 4 min. Light transmission loss through the smoke volume generated under these controlled burning conditions was used to quantify smoke density.

## 4. Conclusions

Ball milling mechanochemistry was used to develop a sustainable, biobased FR through the chemical modification of microcrystalline cellulose with P_2_O₅ under mild process conditions. The reaction with P_2_O₅ was carried out for 1 h, and the recovered powder was washed/centrifuged with deionized water. ICP analysis estimated the phosphorus content in Cel-P to 4.15 wt.%, corresponding to a degree of substitution of about 0.25%. This derivatization ensures a uniform presence of phosphorus on the surface of cellulosic microparticles, improving the thermal resistance of the char formed during cellulose degradation followed by TGA, even under an oxidizing atmosphere. However, the presence of phosphoric acid groups also induces premature cellulose thermal degradation.

The incorporation of Cel-P into PP improved the composite fire performance, as evidenced by a 30% reduction in the pHRR and a decrease in THR in the MLC test, although it unfortunately reduced the ignition time. In smoke density testing, the PP+30-Cel-P composite showed lower smoke opacity than PP+30-Cel but still higher than neat PP. This was attributed to the presence of phosphorylated cellulose, which acts as a phosphorus-based FR known to disrupt the combustion process, shifting it from complete to incomplete combustion and thereby generating more smoke. Additionally, certain phenomena occurring during the test artificially reduce smoke production in PP and PP+30-Cel, such as droplet formation, which decreases the amount of burned material, and the presence of flames, which inhibits smoke formation.

## Figures and Tables

**Table 1:** TGA results for Cel, Cel-P, PP, and related composites, based on thermograms obtained under air and nitrogen, with heating rate of 10 °C.min^−1^.

Composition	Under Air				Under N_2_			
	T_-20 wt.%_ (°C)	T_-40 wt.%_ (°C)	T_degrad_ (°C)	Residue (%)	T_-20 wt.%_ (°C)	T_-40 wt.%_ (°C)	T_degrad_ (°C)	Residue (%)
Cel	305	320	320; 500	0	328	336	345	6
Cel-P	215	276	222; 515; 645	2	215	267	224	35
PP	440	450	455	0				
PP+30-Cel	344	445	330; 460	5				
PP+30-Cel-P	440	455	218; 463	13				

**Table 2:** Results of the mass loss cone calorimeter test at 35 kW.m^−2^.

Composition	TTI (s)	pHRR (kW.m^−1^)	THR (MJ.m^−2^)	pHRR Change (%)
PP	60	725	74	----
PP+30-Cel	48	774	79	+7
PP+30-Cel-P	34	514	62	−30

## Data Availability

The Data not available due to privacy restrictions.
